# Structural Characterization of a Macrocyclic Peptide Modulator of the PD-1/PD-L1 Immune Checkpoint Axis

**DOI:** 10.3390/molecules26164848

**Published:** 2021-08-11

**Authors:** Edyta Zyla, Bogdan Musielak, Tad A. Holak, Grzegorz Dubin

**Affiliations:** 1 Malopolska Centre of Biotechnology, Jagiellonian University, Gronostajowa 7A, 30-387 Krakow, Poland; zyla.edyta@doctoral.uj.edu.pl; 2 Faculty of Chemistry, Jagiellonian University, Gronostajowa 2, 30-387 Krakow, Poland; bogdan.musielak@uj.edu.pl (B.M.); holak@chemia.uj.edu.pl (T.A.H.)

**Keywords:** immunotherapy, immune checkpoint, PD-L1, PD-1, macrocyclic peptides

## Abstract

The clinical success of PD-1/PD-L1 immune checkpoint targeting antibodies in cancer is followed by efforts to develop small molecule inhibitors with better penetration into solid tumors and more favorable pharmacokinetics. Here we report the crystal structure of a macrocyclic peptide inhibitor (peptide 104) in complex with PD-L1. Our structure shows no indication of an unusual bifurcated binding mode demonstrated earlier for another peptide of the same family (peptide 101). The binding mode relies on extensive hydrophobic interactions at the center of the binding surface and an electrostatic patch at the side. An interesting sulfur/π interaction supports the macrocycle-receptor binding. Overall, our results allow a better understanding of forces guiding macrocycle affinity for PD-L1, providing a rationale for future structure-based inhibitor design and rational optimization.

## 1. Introduction

Anticancer therapies relying on immune checkpoint inhibition are considered one of the outstanding achievements of recent years in clinical oncology. This approach targets cancer by activating the patient’s own immune system [1,2]. The first in class drug, ipilimumab, targeting CTLA4 was authorized in 2011, but it is the anti-PD-1 and anti-PD-L1 antibodies that have revolutionized the field [3,4,5,6,7]. Despite its spectacular success, the antibody-mediated inhibition of immune checkpoints does have its limitations. Some of the limitations are related to the pharmacokinetics and penetration of antibodies [8,9,10]. Small molecules offer a promising alternative. Attempts are ongoing in academia and pharma to develop potent inhibitors of immune checkpoints, with the PD-1/PD-L1 axis attracting major attention [11,12]. A number of small-molecule inhibitors (<600 Da) have been reported to date, but none have demonstrated satisfactory cellular activities yet [13]. Macrocyclic peptide inhibitors have shown promising results. In two subsequent patents, Bristol-Myers Squibb have reported three classes of macrocyclic peptides containing modified amino acids (classes I and II) and proteinogenic amino acids only (class III) [14,15]. All the classes contained macrocycles characterized by nanomolar affinities towards PD-L1. We have recently characterized the biological activity and the binding modes of representatives of all three classes [16,17] to guide further development. Interestingly, the crystal structure of peptide 101 representing class III exhibited an unusual bifurcated binding mode [17] which complicated the understanding of interactions guiding the affinity of this class of macrocycles. In this study, we characterize the interaction of a related macrocycle (peptide 104) to demonstrate that the bifurcated binding is not a common feature of this class of compounds. Peptide 104 was selected for the experiments because it is a close structural analog of p101, and both macrocycles are characterized by comparable affinity. Detailed characterization of the binding hot-spots uncomplicated by bifurcated binding mode facilitates rational design and optimization of PD-1/PD-L1 inhibitors.

## 2. Results

### 2.1. Initial Characterization

**Figure 1:**
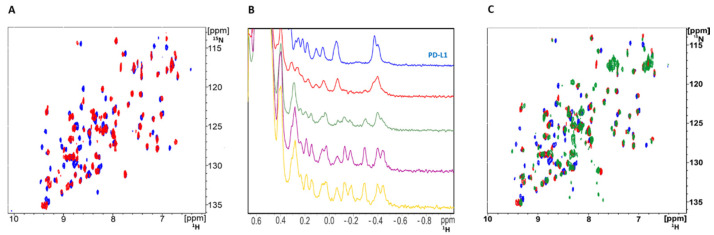
NMR indicates the physical interaction of p104 with PD-L1. (**A**) ^1^H-^15^N HMQC spectra of ^15^N labeled PD-L1 (blue) and the same protein in the presence of p104 (red). (**B**) ^1^H NMR titration of PD-L1 (blue) with peptide p104 in the molar ratios 5:1 (red), 2:1 (green), 1:1 (purple), and 1:2 (yellow). (**A**,**B**) Significant shifts observed in positions of resonance peaks indicate interaction of p104 and PD-L1. (**C**) ^1^H-^15^N HMQC spectra of apo-PD-L1 (blue) and the same protein in the presence of p104 (red) or p101 (green) in the molar ratio 1:1.

Peptide 104 (p104) was synthesized based on the structure disclosed in the patent [18]. The interaction of p104 with PD-L1 was validated using NMR titration; ^15^N labeled PD-L1 was contacted with increasing amounts of p104, and changes in the chemical shifts of ^1^H-^15^N cross-correlation peaks were monitored in a 2D HMQC experiment. Significant shifts of a number of protein-originating cross-correlation peaks indicated the physical interaction of p104 and PD-L1 (Figure 1A). The same conclusion was reached by monitoring the chemical shifts of ^1^H resonances in the aliphatic region of 1D ^1^H-NMR spectra of PD-L1 upon titration with p104 (Figure 1B). No peak doubling was observed at the spectra of the PD-L1/p104 complex, suggesting a single binding conformation. The comparable pattern of peak shifts in the PD-L1/p104 and PD-L1/p101 [17] HMQC spectra compared to the reference spectrum of apo-PD-L1 suggests comparable binding modes of both macrocycles (Figure 1C).

**Figure 2:**
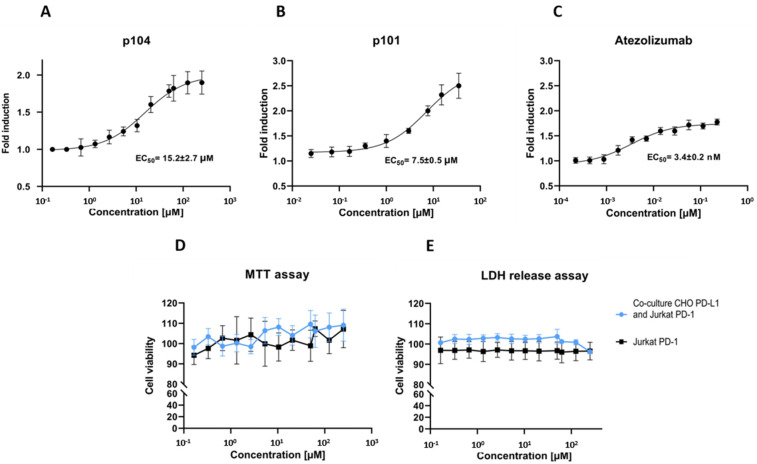
Macrocyclic peptide 104 inhibits PD-1/PD-L1 interaction in the cellular environment. The Jurkat cell line overexpressing PD-1 and carrying the NFAT-driven luciferase reporter was contacted with CHO-K1 cells stably overexpressing the TCR activator and PD-L1, and the activity of the reporter was monitored at varying concentrations of (**A**) p104, (**B**) p101, or (**C**) control antibody (atezolizumab). Atezolizumab and the tested macrocycles increase luciferase production (as determined by the increase in the luminescence level), indicating abrogation of PD-L1-mediated inhibition of TCR-induced Jurkat cell activation. The effect is dose-dependent. Half-maximal effective concentrations (EC_50_) were calculated by fitting the Hill equation to the experimental data. (**D**,**E**) Effect of p104 on cell viability determined with the MTT assay (**D**) and LDH release (**E**). The data are shown as mean ± SD from three independent experiments normalized to the control (vehicle-treated cells).

To evaluate the ability of p104 to dissociate the PD-1/PD-L1 interaction, we used a cell-based interaction assay. In the assay, the Jurkat lymphocyte-like cell line overexpressing PD-1 and carrying an NFAT-driven luciferase reporter is contacted with CHO-K1 cells stably overexpressing the TCR activator and PD-L1. In the absence of PD-1/PD-L1 interaction inhibitors, PD-1 signaling mitigates the stimulating effect of TCR induction, and the reporter level is low. The FDA-approved therapeutic monoclonal antibody targeting PD-L1 (atezolizumab) dose-dependently dissociates the PD-1/PD-L1 complex resulting in increased reporter expression (Figure 2C). The same is true for p104, demonstrating its ability to dissociate the PD-1/PD-L1 complex, although with EC_50_ values above those characterizing the antibody effect (Figure 2A). EC_50_ characterizing p104 (15.2 ± 2.7 µM) is higher compared to that of p101 (7.5 ± 0.5 µM; [17]), which reflects the difference in the affinity of the tested macrocycles towards PD-L1 (380 nM vs. 120 nM, respectively). The macrocycle is not significantly toxic to the cells at concentrations inducing the biological effect (Figure 2D,E).

### 2.2. Structural Basis of PD-L1 Interaction with p104

To elucidate the interactions guiding the affinity of the macrocyclic peptide p104 and PD-L1, we co-crystallized the complex. The extracellular distal domain of PD-L1 was expressed in *E. coli*, refolded, and purified to homogeneity. P104 was added at three-fold molar excess. Crystallization trials were performed in commercially available buffer sets. Diffraction data were collected using the obtained crystals. The structure was solved by molecular replacement at 1.9 Å resolution (Table 1).

The asymmetric unit contains a single protein/macrocycle complex. The distal extracellular domain of PD-L1 used for crystallization and the macrocycle ligand are well described by their respective electron densities, save for several side chains. The N- and C-termini of the macrocycle are characterized by temperature factors higher than the average values characteristic for the protein molecule. The uneven distribution of temperature factor values characterizing the atoms constituting the macrocycle is related to crystal packing. The central part of the macrocycle is stabilized by interactions with symmetry-related molecules, while the N- and C-termini point into a solvent channel.

The macrocyclic inhibitor folds into a β-hairpin additionally stitched at the free ends by the cyclizing bond. The macrocycle binds almost perpendicular to the strands constituting the C, C’, G, and F β-sheet of the PD-L1 IgV domain, docking at its slightly concave hydrophobic surface. The primary interactions are contributed by G and F strands, while C and C’ strands provide less significant interactions.

**Figure 3:**
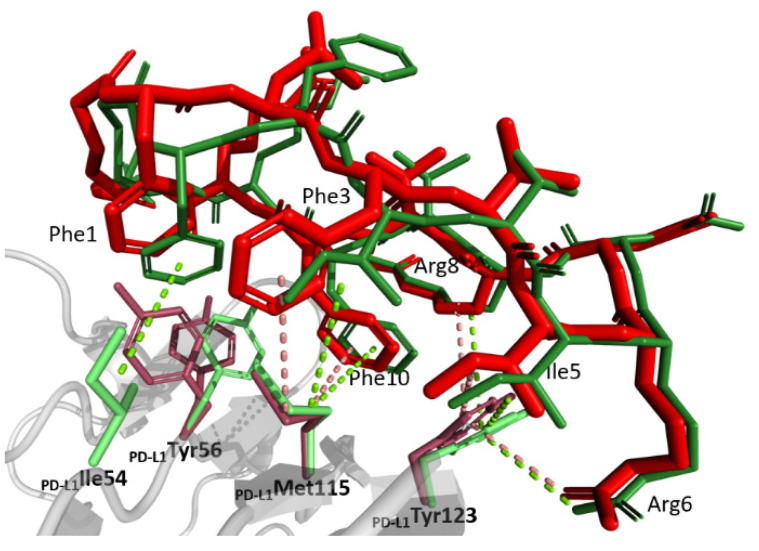
Comparison of the binding modes of p104 and p101 at the surface of PD-L1 receptor. Peptide 104 (red) and peptide 101 (green, single conformation; the second conformation is not shown for clarity) binding to PD-L1 (gray; residues relevant for interactions are shown in pink and light green, respectively, for p104- and p101-containing structures). Major interactions are depicted with dotted lines.

The macrocycle exposes a large hydrophobic patch composed of π-stacked sidechains of _104_Phe1 and _104_Phe3, _104_Ile5, the proximal atoms of _104_Arg8 and _104_Phe10, which complements the relatively flat hydrophobic patch at the surface of PD-L1 (Figure 3). This indicates that the macrocycle sidechains reside in shallow grooves rather than classical pockets. The first two of the above macrocycle residues dock at a shallow groove made of sidechains Ile54, Tyr56, Met115, C_β_ of Ser117, and Ala121 of PD-L1. The sulfur/π interaction between _104_Phe3 and Met115 strengthens the binding. The sidechains of _104_Ile5 and _104_Phe10 dock at an adjacent shallow groove composed of sidechains Met115, Ala121, proximal atoms of Arg113, and the sidechain of Tyr123 with the most significant interactions involving alkyl-π interaction of Met115 and _104_Phe10 and T-stacking of the latter residue and Tyr123 sidechain. The _104_Phe10 pocket is completed by the sidechain of Glu58, which provides oxygen-π interactions with the inhibitor. The proximal atoms of _104_Arg8 top the above binding site providing additional hydrophobic contacts with the sidechain of Tyr123, while the guanidinium moiety stacks with that of Arg113. Residual hydrophobic interactions are provided by the distal residue of the macrocycle and Val76.

Polar contacts are less developed compared to the hydrophobic surface. _104_Arg6 contributes two direct hydrogen bonds with the carboxyl of Asp122 and the carbonyl oxygen of Tyr123. A water-mediated interaction between the carbonyl oxygen of _104_Val9 and the carboxyl of Glu58 further supports binding.

The β-hairpin structure of the macrocycle requires that hydrophobic residues of _104_Leu2, _104_Val4, and _104_Val9 are solvent-exposed. Such unfavorable solvent contacts are partially compensated by simultaneous exposure of polar residues: _104_Asp7, _104_Arg8, and _104_Arg11 and the carbonyl oxygens of proximal residues within the macrocycle.

### 2.3. Rationale of PD-1/PD-L1 Interaction Inhibition

The overlay of the structures of PD-L1 in complexes with p104 determined in this study and with PD-1 determined previously [19] reveals that the binding sites at the surface of PD-L1 largely overlap. The higher affinity of p104 towards PD-L1 compared to the affinity of PD-1 towards PD-L1 determines the competitive mode of inhibition. In fact, p104 mimics the arrangement of strands G and F of PD-1. However, because of the opposite direction of the mainchain in both molecules, no significant correspondence of sidechain accommodating pockets is seen apart from _104_Phe10 occupying a pocket resembling that occupied by _PD-1_Ile134. Residues _104_Phe3, _104_Ile5, and _104_Arg6 occupy the same binding groove as _PD-1_Leu128, _PD-1_Ile126, and _PD-1_Tyr68; however, neither of the residue binding sites correspond directly to each other. Minor adjustments in the disposition of the sidechains of PD-L1 are seen between the structures of the p104 and PD-1 complexes. However, the adjustments are insignificant compared to the differences in the structures of the binding partners. Interestingly, binding of both PD-1 and p104 involves comparable adjustments of the PD-L1 surface compared to the apo-structure.

### 2.4. Comparison of the Binding Modes of p104 and p101

The primary goal of this research was to clarify whether the unusual bifurcated binding mode determined previously for peptide 101 [17] and complicating the understanding of class III peptide interactions with PD-L1 was characteristic for this group of macrocycles or whether it was p101 structure specific. To this end, p104 was selected, which differs from p101 only at positions 2 and 3 (_104_Leu2Phe3 vs. _101_Phe2Ile3) and is characterized by comparable affinity (380 vs. 120 nM). The structure of p104 in complex with PD-L1 determined in this study shows no indication of macrocycle bifurcation, demonstrating that such a phenomenon is characteristic either for p101 only or for the particular crystal lattice characterizing the previously reported p101 structure. The orientation of p104 at the surface of PD-L1 is best described as an averaged orientation of two binding modes observed in the bifurcated structure of p101 (Figure 3). The reduced affinity of p104 compared to p101 is difficult to explain on a structural basis, especially that _104_Phe3 seems to better fit the relevant groove compared to _101_Ile3.

## 3. Discussion

Macrocyclic peptides are one of several classes of non-antibody PD-1/PD-L1 interaction inhibitors capable of dissociating the protein–protein complex and relieve T-cell anergy, at least in an in vitro setting. While they are not valid clinical candidates at the moment, the investigation of interactions guiding the affinity of these compounds facilitates rational design and optimization of novel inhibitors. Prior to this study, understanding of the structural basis guiding the affinity of class III macrocycles containing only proteinogenic amino acids has been limited by the unique bifurcated binding mode of its only representative (p101) for which the experimental structure was available in complex with PD-L1 [17]. This study demonstrates that p104 binds in the same region of PD-L1 in a single conformation resembling an “averaged” conformation of the two binding modes observed for p101. The NMR analysis supports the conclusion that, in a solution, p104 is characterized by a single binding pose at the surface of PD-L1. Such results allow a clear description of interactions guiding the affinity of class III macrocycles.

Hydrophobic interactions centered around Tyr56, Met115, and Tyr123 predominate the binding surface. Residual polar contacts supplement the interaction. The binding region of the macrocycle coincides with that of PD-1, but despite the comparable overall binding mode, only a single residue of the inhibitor (_104_Phe10) directly mimics the binding of PD-1 residues. Other residues of the inhibitor use the same binding grooves as those of PD-1, but with a different set of detailed interactions.

The binding surface of p104 also coincides with that of representatives of macrocycles from group I (p57) and group II (p71), but the detailed interactions are yet distinct. This demonstrates that the relatively flat binding surface of PD-L1 is a landscape for versatile interactions of new diverse classes of compounds yet to be discovered. By contributing to a better understanding of molecular interactions, our study facilitates the design of novel PD-1/PD-L1 interaction inhibitors.

Macrocyclic peptide 104 effectively blocks PD-1/PD-L1 interaction at the cell surface. The biological activity of p104 determined in a cell-based assay is weaker than that of p101, corresponding to the lower affinity of the former macrocycle towards PD-L1. However, p104 was selected for the experiments not because of its superior activity, but as a close structural analog of p101. Yet, p104 still exerts its biological effect at concentrations at which cytotoxicity is not an issue. In the light of cytotoxicity associated with a number of low molecular weight PD-1/PD-L1 interaction inhibitors [12], the low cytotoxicity characterizing macrocyclic inhibitors [16,17] constitutes a major advantage. The availability of structural information and correlation of PD-L1 affinity and biological effects of macrocycles offers an additional advantage for optimization. It remains to be determined, however, whether the macrocyclic scaffold can be optimized for biological activity comparable to that of antibodies.

## 4. Materials and Methods

### 4.1. Protein Expression, Purification, and Quality Evaluation 

The distal extracellular domain of PD-L1 (amino acids 18–134) was expressed from a pET-21b vector in *E. coli* strain BL21. The bacteria were cultured overnight at 37 °C in LB medium supplemented with a selection antibiotic (100 µg/mL ampicillin). The culture was diluted 25-fold and incubated at 37 °C until the OD_600_ reached 0.6 when protein expression was induced with 1mM IPTG. The expression was continued for 6h at 37 °C. Inclusion bodies were released by sonication and collected by centrifugation. The inclusion bodies were washed four times with 50 mM Tris-HCl pH 8.0 containing 200 mM NaCl, 10 mM EDTA, 10 mM 2-mercatopethanol, and 0.5% Triton-X. They were resuspended in denaturation buffer containing 6 M GuHCl, 200 mM NaCl, and 10 mM 2-mercaptoethanol in 50 mM Tris-HCl pH 8.0 at 4 °C for about 16 h using a tube roller. The solution was clarified by high-speed centrifugation, and the protein of interest was refolded by drop-wise dilution in 100 mM Tris-HCl pH 8.0 containing 1M L-arginine, 0.25 mM oxidized glutathione, and 0.25 mM reduced glutathione. The refolded protein was dialyzed 3 times against 10 mM Tris-HCl pH 8.0 containing 20 mM NaCl and purified by size-exclusion chromatography using Superdex 75 in the same buffer. The purity of refolded PD-L1 was estimated by SDS-PAGE, and folding was determined by ^1^H NMR.

### 4.2. NMR

All spectra were recorded in 10 mM Tris-HCl pH 8.0 and 20 mM NaCl containing 10% (*v*/*v*) of D_2_O to provide the lock signal. The spectra were recorded using a Burker Avance III 600 MHz spectrometer. PD-L1 folding was routinely determined by ^1^H NMR. PD-L1/p104 complex formation was evaluated by ^1^H NMR and ^1^H-^15^N SOFAST-HMQC NMR of ^15^N isotopically enriched PD-L1 by monitoring protein-associated resonance signal shifts upon macrocycle titration.

### 4.3. Crystallization of the PD-L1 Complex with p104

PD-L1 (5 mg/mL) and p104 were mixed at a 1:3 molar ratio. Screening for crystallization conditions was performed using commercially available screening buffers. The sitting drop vapor diffusion method was utilized. The initially obtained conditions were optimized by standard methods. Diffraction quality crystals were obtained at 20 °C from 0.1 M sodium citrate containing 1.95 M ammonium sulfate pH 6.2. The crystals were soaked in a cryoprotectant solution containing reservoir buffer with 25% glycerol and flash cooled in liquid nitrogen. The data were collected at BESSY II 14.1 beamline operated by Helmholtz-Zentrum Berlin für Materialen und Energie (HZB).

### 4.4. Structure Determination and Refinement

The data were processed using XDS and scaled using Scala contained in the CCP4 package [20,21]. The structure was determined by molecular replacement performed with Phaser and using apo PD-L1 as a search model (PDB ID 5O45) [22]. Model building in the resulting electron density maps was performed using WinCoot software [23]. Refinement was achieved using Refmac5 [24]. The data collection and refinement statistics are summarized in Table 1. The coordinates were deposited at the Protein Data Bank under accession number 7OUN. The structure was analyzed using Discovery Studio, and molecular graphics were prepared using PyMol [25,26].

### 4.5. Cell Culture

CHO-K1 expressing the TCR activator and PD-L1 and Jurkat T cells carrying an NFAT-dependent luciferase gene and overexpressing PD-1 were obtained from Promega. The cells were cultured in RPMI 1640 medium supplemented with 10% FBS with hygromycin B (50 µg/mL) and G418 (250 µg/mL) at 37 °C with 5% CO_2_.

### 4.6. Cell-Surface PD-L1/PD-1 Immune Checkpoint Interaction Assay

24 h before the experiment, the CHO-K1 cells were seeded on a 96-well plate in an amount of 10,000 cells/well. 2.5-fold serial dilutions of p104 were prepared in DMSO followed by 1000× dilution in the assay buffer (RPMI 1640, 1% FBS). The culture medium was replaced with the assay buffer containing 0.02–250 µM of p104. The Jurkat cells in the amount of 50,000 cells/well were added and co-cultured for 3 h in standard conditions. Luminescence was determined using Bio-Glo Assay reagent (Promega, Madison, WI, USA) following manufacturer protocol. Data were normalized at vehicle-treated control.

### 4.7. Cytotoxicity Assay

The cytotoxic effect of the tested macrocycle against the Jurkat cells alone or in the co-culture with the CHO-K1 cells was evaluated. The cells were incubated in a medium containing 0.02–250 µM p104 for 24 h. Released cytosolic LDH was determined using a Pierce LDH Assay Kit (Thermo Scientific, Boston, MA, USA) according to the manufacturer’s protocol. For the MTT assay, the macrocycle-pretreated cells were incubated with 0.45 mg/mL MTT for 2 h at 37 °C. The medium was discarded and replaced with DMSO:methanol (1:1). The samples were incubated for 20 min at RT with pipetting to dissolve the formazan crystals. Absorbance was determined at 550 nm. The data were normalized at vehicle-treated control.

## Figures and Tables

**Table 1:** Data collection and refinement statistics.

**Data Collection:**	
Wavelength (Å)	0.9184
Space group	*P* 2 3
**Cell Dimensions:**	
a,b,c (Å)	86.5 86.5 86.5
α,β,γ (°)	90.0 90.0 90.0
Resolution range (Å)	61.1–1.9
R_merge_	0.091 (1.576)
I/σ(I)	18.1 (2.6)
Completeness (%)	100.00 (100.00)
Unique reflections	17,309
CC_1/2_	1.000 (0.666)
**Refinement Statistics:**	
No. of reflections	480,286
R_work_/R_free_	0.232/0.246
Wilson B-factor	40.2
No. of atoms	1098
Protein	1061
Water	37
Ramachandran favored (%)	91.0
Ramachandran allowed(%)	5.7
Ramachandran outliers (%)	3.3
**R.m.s Deviation**	
Bond lengths (Å)	0.016
Bond angles (°)	2.262

Data in parentheses are for the highest resolution shell.

## Data Availability

The data presented in this study are openly available in PDB database, ID: 7OUN.

## Sample Availability

Samples of the compound p104 are available from the authors.
